# Being right, but losing money: the role of striatum in joint decision making

**DOI:** 10.1038/s41598-018-24617-3

**Published:** 2018-04-30

**Authors:** M. I. Ruissen, S. Overgaauw, E. R. A. de Bruijn

**Affiliations:** 1 Institute of Psychology, Leiden University, Leiden, The Netherlands; 2 Leiden Institute for Brain and Cognition (LIBC), Leiden, The Netherlands

## Abstract

Joint decision-making entails that you sometimes have to go along with the other’s choice even though you disagree. In this situation, a resulting negative outcome may, however, elicit a feeling of satisfaction and an impulse to say “I told you so”. Using fMRI, we investigated the neural correlates of this complex process comprised of both positive and negative outcomes. During a social visual search task, 19 participants gave their advice to a co-actor who then made the decision resulting in a mutual loss or gain. This design allowed direct comparisons of situations that resulted in the same monetary outcome but that differed with respect to the correctness of the initial advice of the participant. Increased striatal activations were found for gains compared to losses and for correct compared to incorrect advice. Importantly, ROI analyses also showed enhanced striatum activation for monetary losses that were preceded by correct compared to incorrect advices. The current study therefore suggests that reward-related neural mechanisms may be involved when being right even in situations that end in monetary losses.

## Introduction

Sitting in the passenger’s seat can be an excruciating experience for several reasons. One that many people can relate to is a disagreement with the driver about the route to take. As a passenger, you can merely voice your opinion and hope that the driver will trust your judgment. However, an interesting phenomenon occurs when the driver ignores your advice and it turns out that you were right after all. Although the decision is likely to result in negative consequences such as adding crucial minutes to your travel time, a feeling of satisfaction, pride, or even an impulse to say “I told you so” may nevertheless be experienced. Here, we want to investigate if and how this process that is a mix of both positive and negative outcomes is reflected in neural activation in areas associated with reward processing.

Initial neuroimaging studies on reward processing mainly focused on primary (e.g., food; crucial for survival) and secondary rewards (e.g., money; rewarding due to learned associations) in individual contexts [see^1^ for a review]. These studies have demonstrated a crucial role for the striatum (specifically the nucleus accumbens; NAcc) in the processing of reward information, such as reward expectation and the role of motivation in retrieving a reward^2,3^. More recent studies in humans have also shown that similar processes and brain areas are involved in socially relevant types of rewards such as social approval^4,5^, donation giving^6^, following advice^7,8^ or interacting with a liked person^9–11^.

Both social and non-social decisions may be driven by motivational processes incorporating the expected value of a certain outcome, specifically in case of secondary rewards^1,3,12,13^. However, in daily life this value may not be that straightforward to determine, especially as outcomes may depend on the social context as well as the behavior of another person. In a previous study^14^, we demonstrated that the striatum responds in a context-specific manner with similar activation patterns for own correct actions and observed correct actions from a partner in a cooperative context as well as for observed errors in a competitive context, all resulting in a monetary gain. Additionally, the relative prosperity between people is known to play a role. Tricomi *et al*.^15^. showed for example that people have inequality-averse social preferences leading to increased reward-related activity when the underdog receives a monetary endowment. This effect can be explained by concerns for reciprocity, because it even holds when it concerns a monetary reward for an unknown other.

In addition, several studies have shown differential activation patterns in the striatum when performing better than an unknown other [see e.g.^16-18^]. Fliessbach and colleagues^17^, for example, demonstrated increased striatal activity in response to relative reward inequity, i.e., in comparison to another participant, while absolute reward remained unchanged. More recently, Lindner *et al*.^18^. showed that striatum was responsive to objective performance differences in a quiz paradigm. Activation was specifically increased for situations where participants gave a correct answer when the majority had failed to do so and emphasizes the relevance of personal performance. The striatum thus seems to play an important role in social reward processing by coding both contextual information and outcome value.

The current fMRI study aims at understanding the sensitivity of reward-related brain areas to social contextual variations further by specifically investigating whether losses of reward are associated with striatal activations merely because of the rewarding effect of being right. To this aim, we developed a social visual search paradigm in which a co-actor, who was seated outside the scanner room, had to make the decision that could result in a mutual gain or loss. Crucially, this co-actor made the decision by either following or ignoring the advice from the participant lying in the scanner. This design allowed us to directly compare situations that resulted in the same absolute monetary outcome (i.e., winning or losing 10 eurocents) but differed with respect to the correctness of the initial advice. We were particularly interested in the situation in which the co-actor ignored the participant’s correct advice resulting in an equal monetary loss for both (i.e., they both lose 10 eurocents). Even though the reward outcomes are the same for the comparisons of interest, we expect striatum to code both the initial correctness and absolute reward outcome, as reflected in relatively increased striatal activations for negative outcomes preceded by a correct compared to an incorrect advice.

## Methods

### Participants

Twenty-one right-handed healthy volunteers participated in the study. Two participants were excluded because of excessive head movement (>3 mm in any direction) resulting in a total of 19 participants (12 females, mean age = 21.8 years, SD = 2.4). Based on a self-report screening questionnaire participants who did not report any current medical or psychiatric disorders were included in the study. Other exclusion criteria were use of medication, excessive smoking, excessive alcohol use, and use of drugs. Participants received 20 euros for their participation and an additional bonus earned during the experiment. All participants provided informed consent. The study was approved by the Leiden University medical ethics committee, and in accordance with the Declaration of Helsinki.

### Task and procedure

Two participants would come to the lab to perform a social visual search paradigm. Unknown to the real participant, the other person was a confederate whose behavior would be simulated by the computer. Seven students from our department volunteered as confederate (5 females). We aimed to match gender, but this was not possible in three cases. Because of the homogeneous student sample, we did not match other characteristics (e.g. race, age). After verbal and written task instructions were given, participants practiced the task together outside the scanner. The practice session consisted of 10 trials. During this practice session, the displayed feedback was based on the actual decisions of the confederate. During the test session in the scanner, however, decisions of the confederate were generated by the computer to ensure equal frequency distributions: In approximately half of the trials the decision was in agreement with the participant’s response, in the other approximate half of the trials the confederate did not agree.

**Figure 1:**
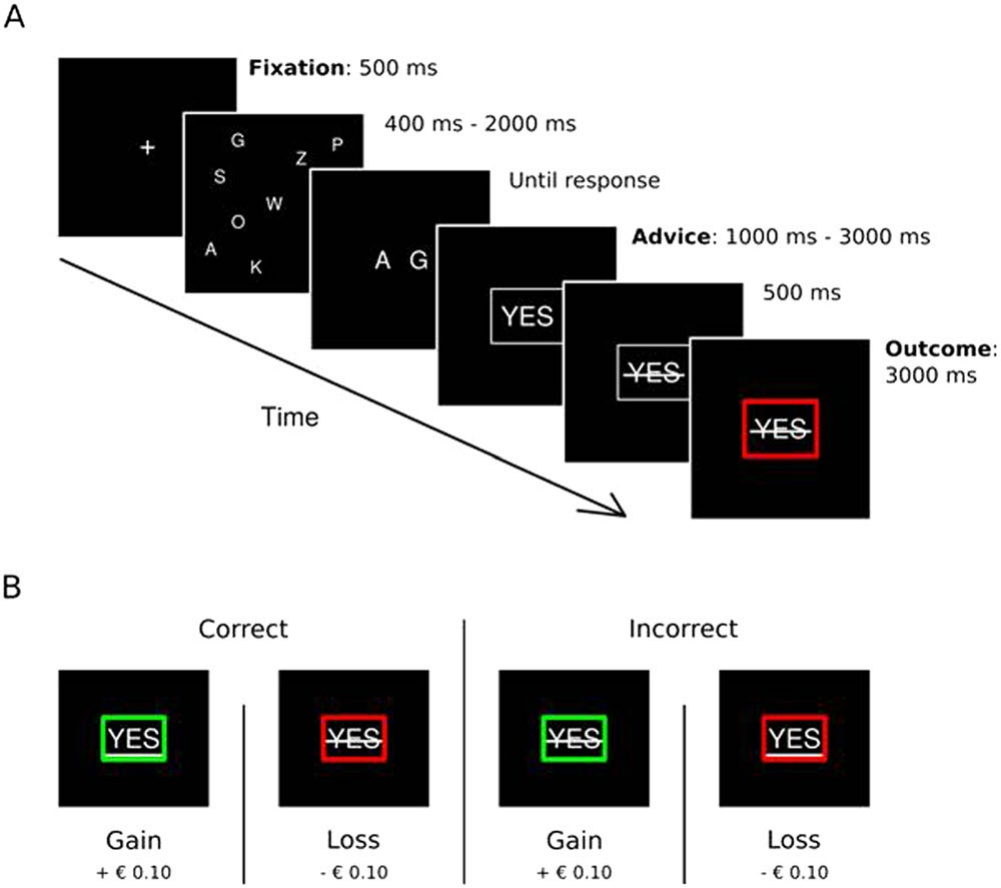
“I told you so” task. (**A**) Example of a correct trial with a negative outcome. (**B**) All 4 possible outcomes. The participant could be correct or incorrect in his/her advice and the outcome based on the co-actors decision could be a monetary gain or loss. If the co-actor agreed with the advice of the participant, the advice (‘YES’ or ‘NO’) was underlined. In case the co-actor disagreed with the advice, this was shown as a strikethrough. Gains were visualized by a green box, and losses by a red box. Incorrect final decisions resulted in a 10 eurocents loss, and correct trials were rewarded by a 10 eurocents gain.

Figure 1 depicts a schematic representation of the task. The task started with a fixation cross displayed for 500 ms followed by a mix of 12 random characters displayed for 400–2000 ms, depending on the participant’s performance. To keep the task challenging but not too difficult, we aimed for an accuracy rate of around 60–65% [see 14] by dynamically increasing or decreasing the stimulus duration with 20 ms based on the participant’s performance. Next, two characters were displayed (until response) and the participant had to indicate whether these two characters had been present in the previously presented mix. The participant could respond by pressing one of eight buttons. The buttons were distributed over two hands such that each finger of each hand was above one button. The buttons on one hand represented a yes-response, and the buttons on the other hand represented a no-response (counterbalanced). Moreover, participants could indicate how certain/confident they were (on a 4-point scale: absolutely certain, very certain, not so certain, very uncertain) by responding with different fingers. This was done to keep participants engaged in the task and also served as an offline check of their engagement (i.e., certainty should be related to correctness). Participants were asked to use their index finger when they were most certain about their response and the other fingers when they were less certain, until the little finger indicating the least certain response.

After participants had given a response, the answer was displayed with a jittered duration between 1000–3000 ms using the Dutch term *wel* in capitals for a yes-response and the Dutch term *niet* in capitals for a no-response. The font size in which the answer was displayed ranged from 16–40 px and reflected their level of certainty with larger font sizes indicating more certainty. Participants were told that the other participant outside the scanner with whom they performed the task would then make the final decision based on the other participant’s own judgment as well as the advice of the participant including confidence information.

The final decision was displayed for 500 ms by underlining the participant’s choice in case of agreement and a line through the participant’s choice in case of disagreement. Next, the outcome was displayed for another 3000 ms by a green or red frame around the stimulus for respectively correct and incorrect final decisions (visual angle = 18.5 × 13.8°). The test session consisted of 200 trials and was divided in four blocks of 50 trials. The entire duration of the experiment was around 35 minutes. Participants were informed that they would start with a bonus of 4 euros and together earned 10 eurocents for each correct final decision and lost 10 eurocents for each incorrect final decision. They would share their earned bonus equally at the end of the experiment. The current bonus status was shown to the participants between two consecutive blocks of trials.

The task was presented using E-prime software (Psychological Software Tools Pittsburgh, PA, USA) on a 19-inch monitor with a minimum acceptable refresh rate of 39 Hz and a maximum acceptable refresh rate of 201 Hz.

### Data acquisition

MRI scans were obtained with a Philips 3.0 Tesla MRI scanner at the Leiden University Medical Center. Foam inserts that surrounded the head restricted head motion. Functional scans for the task were acquired during four runs with T2*-weighted echo-planar imaging (EPI). The first two volumes of each run were discarded to allow for equilibration of T1 saturation effects. After the functional scanning the following settings have been used: TR = 2.2 s, TE = 30 ms, sequential acquisition, 38 slices, slice thickness = 2.75 mm, Field of View (FOV) = 220 × 220 × 114.68 mm.

The experimental task was projected on a screen, which was visible to participants through a mirror. Data were analyzed using SPM8 (Wellcome Department of Cognitive Neurology, London). The following pre-processing steps were used: correction for slice timing acquisition and rigid body motion, spatial normalization to T1 templates (MNI305 stereotaxic space)^19^ using a 12-parameter affine transform together with a nonlinear transformation involving cosine basis functions and resampling of the volumes to 3 mm voxels. Functional scans were smoothed with an 8 mm FWHM isotropic Gaussian kernel. Translational movement parameters exceeded 1 voxel (<3 mm; total drift from the reference volume) in case of two participants, leading to the exclusion of these participants from further analyses. The participants who were included in the final analyses had a mean and maximum head movement of 0.14 and 1.44 mm; head movement never exceeded 3 mm in any direction for any subject or scan.

All events were time locked to the onset of the outcome screen. The trial functions were used as covariates in a general linear model; along with a basic set of cosine functions that high-pass filtered the data. The least-squares parameter estimates of height of the best fitting canonical HRF for each condition were used in pair-wise contrasts. The resulting contrast images, computed on a subject-by-subject basis, were submitted to group analyses. Task-related responses were considered significant if they exceeded a FWE voxel level threshold of *p* < 0.05^18^.

### fMRI data analysis

Statistical analyses were performed on individual participant’s data using the general linear model in SPM8. The onset of ShowLetters, GiveAdvice, Showadvice, FinalChoice, and Outcome were modeled as separate events in an event-related design. Outcome was labeled as: Correct-Gain, Incorrect-Gain Correct-Loss, and Incorrect-Loss. The onset of the Outcome display was modeled as zero duration events. The modeled Outcome events based on performed trials were used as covariates of interest in a general linear model along with a basic set of cosine functions that high-pass filtered the data and a covariate for run effects. The least-squares parameter estimates of height of the best-fitting canonical HRF for each condition were used in pair-wise contrasts.

The four different events were included in a full factorial design including the within-subjects factors Advice (Correct > Incorrect) and Outcome (Gain > Loss). We were interested in the main effects of Advice, Outcome, and possible interaction effects. Moreover, we performed conjunction analyses in order to test for overlapping significant voxels in the main effects of Advice (Correct > Incorrect) and Outcome (Gain > Loss) that did not show any significant interaction effects. Task-related responses were considered to be significant if they exceeded a voxel-based family-wise error correction for multiple comparisons (FWE; *p* < 0.05). We used the Marsbar toolbox for use with SPM8^20^ to perform ROI analyses focused on brain regions of a priori interest (striatum). ROI masks of anatomical striatum (NAcc in specific) clusters for left and right striatum were extracted from the Harvard-Oxford subcortical atlas (http://www.fmrib.ox.ac.uk/fsl/). The parameter estimates we extracted from these clusters for the whole-brain contrast Gain versus Loss have been used to perform paired t-tests for the pairs: Correct-Gain > Incorrect-Gain, and Correct-Loss > Incorrect-Loss. We were specifically interested in these pairs as they allow testing for the unique contribution of being right while keeping the monetary outcome constant. All brain coordinates are reported in MNI atlas space and represent the peak voxels.

## Results

### Behavioral results

Participants responded correctly on 62% of the trials, indicating that the dynamic adjustment of display duration was effective. Table 1 shows the occurrence of each trial type and mean confidence rating.

**Table 1:** Mean occurrence of each trial type with standard deviations in parentheses.

	Correct		Incorrect	
	Gain	Loss	Gain	Loss
	Mean (SD)	Mean (SD)	Mean (SD)	Mean (SD)
Percentage of trials (%)	31 (1.61)	31 (1.44)	19 (1.90)	19 (1.18)
Mean confidence rating (1–4)	2.26 (0.57)	2.29 (0.52)	2.02 (0.48)	2.02 (0.50)

**Figure 2:**
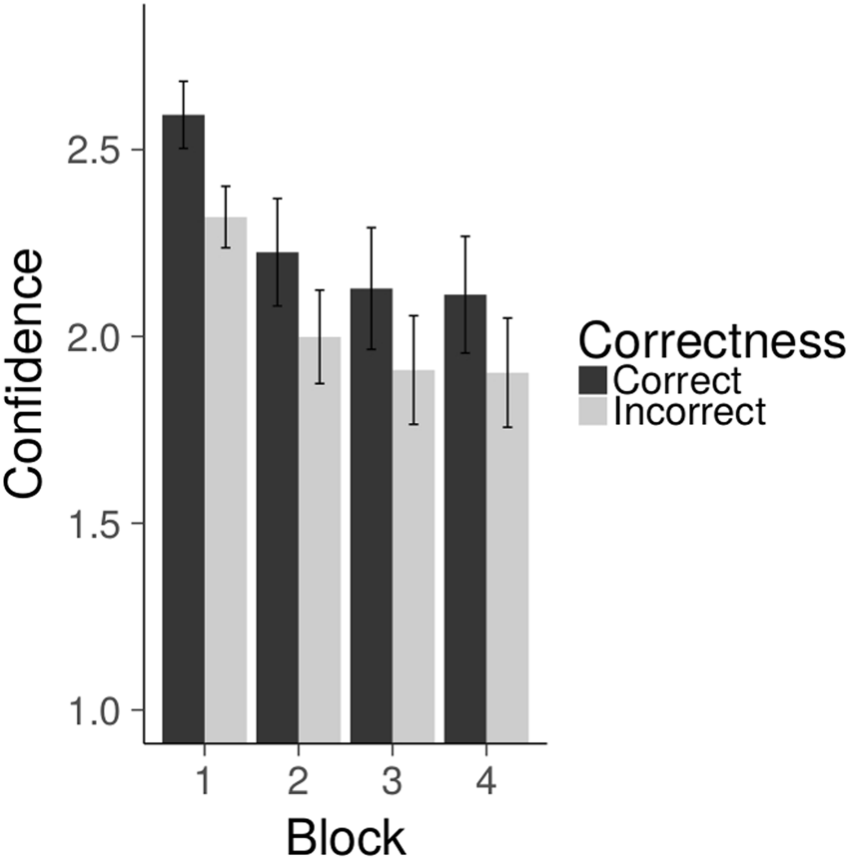
Mean confidence ratings for correct and incorrect advices across the four blocks of the experiment.

To explore how confidence ratings were related to performance, we grouped the ratings in low and high confidence (low confidence: 1 or 2, high confidence: 3 or 4). The difficulty of the task was reflected in the finding that 35.7% of the trials were high confidence scores. Importantly, of these high confidence scores, 70.4% of the trials were correct, suggesting that confidence and performance were related. To check whether this effect remained during the entire duration of the experiment, mean confidence ratings of each of the four experimental blocks were calculated and a Correctness x Outcome x Block ANOVA was conducted. There was a main effect of Correctness [F(1,18) = 23.79, *p* < 0.001]. Participants were more confident on correct trials (M = 2.3) than on incorrect trials (M = 2.0) (see Fig. 2), showing that participants paid attention and were engaged with the task. The main effect of outcome was not significant, but the main effect of Block was [F(3,54) = 10.54, *p* < 0.001], indicating that confidence ratings decreased during the experiment. This finding is the direct result of the stair-casing procedure applied, which limited learning effects on the task by increasing difficulty (i.e., shorter stimulus presentation durations). Importantly, the Correctness x Block and the Correctness x Block x Outcome interaction effects were not significant (both *p*s > 0.098). Thus, differences in confidence ratings between correct and incorrect trials were comparable across the four blocks, suggesting that participants remained motivated during the course of the entire experiment.

### fMRI results

**Figure 3:**
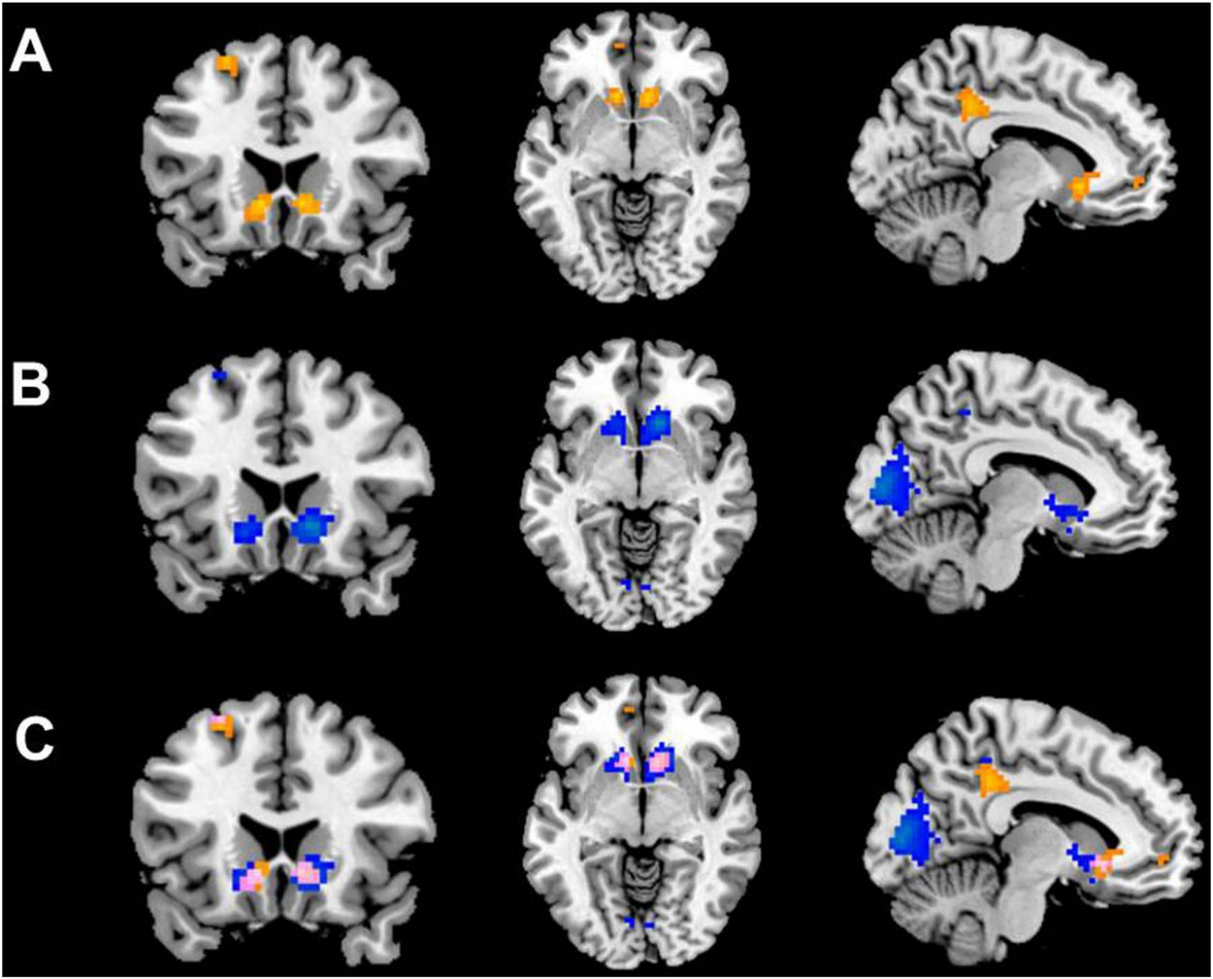
(**A**) Whole-brain contrast (full factorial design) for the main effect of Correct > Incorrect for N = 19 (FWE corrected, *p* < 0.05); (**B**) Whole-brain contrast (full factorial design) for the main effect of Gain > Loss for N = 19 (FWE corrected, *p* < 0.05); (**C**) Conjunction analysis for the main effects Correct >Incorrrect, and Gain > Loss (FWE corrected, *p* < 0.05).

The whole brain contrast involved a 2 (Advice: Correct > Incorrect) x 2 (Outcome: Gain > Loss) ANOVA using a full factorial design. The main effect of Advice (Correct > Incorrect) revealed activation in the cingulate gyrus, superior frontal gyrus, left striatum, and right striatum (N = 19; FWE-corrected, *p* < 0.05; see Fig. 3a, and Table 2). The main effect of Outcome (Gain > Loss) resulted in activation in four task-related areas (N = 19; FWE-corrected, *p* < 0.05): left striatum, right striatum, cuneus, and precuneus (see Fig. 3b, and Table 2). No significant interaction effects were found. However, because of overlapping brain regions in both main effects, we performed a conjunction analysis in order to investigate shared common processes for Advice and Outcome. Results showed two overlapping brain areas (see Fig. 3c): left striatum (33 voxels; MNI = −12, 17, −8; FWE-corrected, *p* < 0.05), and right striatum (34 voxels; MNI = 9, 20, −5; FWE-corrected, *p* < 0.05).

**Table 2:** All brain coordinates for the whole-brain contrasts Correct > Incorrect, and Gain > Loss in a full factorial design for N = 19 (FWE corrected, *p* < 0.05).

Contrast	Region	MNI (x, y, z) coordinates	*Z*-value	Volume (=k_E_ value in SPM)
Correct > Incorrect	Cingulate Gyrus	−3, −37, 37	6.33	188
	Left striatum	−9, 20, −5	5.79	39
	Right striatum	9, 20, −5	5.73	34
	Superior Frontal Gyrus	−24, 23, 55	5.71	37
Gain > Loss	Cuneus	0, −85, 13	7.69	421
	Right striatum	12, 20, −5	6.39	109
	Left striatum	−15, 20, −8	5.63	53
	Precuneus	6, −40, 46	5.28	33

### ROIs

For the ROI analyses we focused on the parameter estimates of the left and right anatomical striatal clusters for the whole-brain contrast Gain versus Loss. Our main interest was to disentangle subtle differences in striatal activation following the four different events: Correct-Gain, Correct-Loss, Incorrect-Gain, Incorrect-Loss.

**Figure 4:**
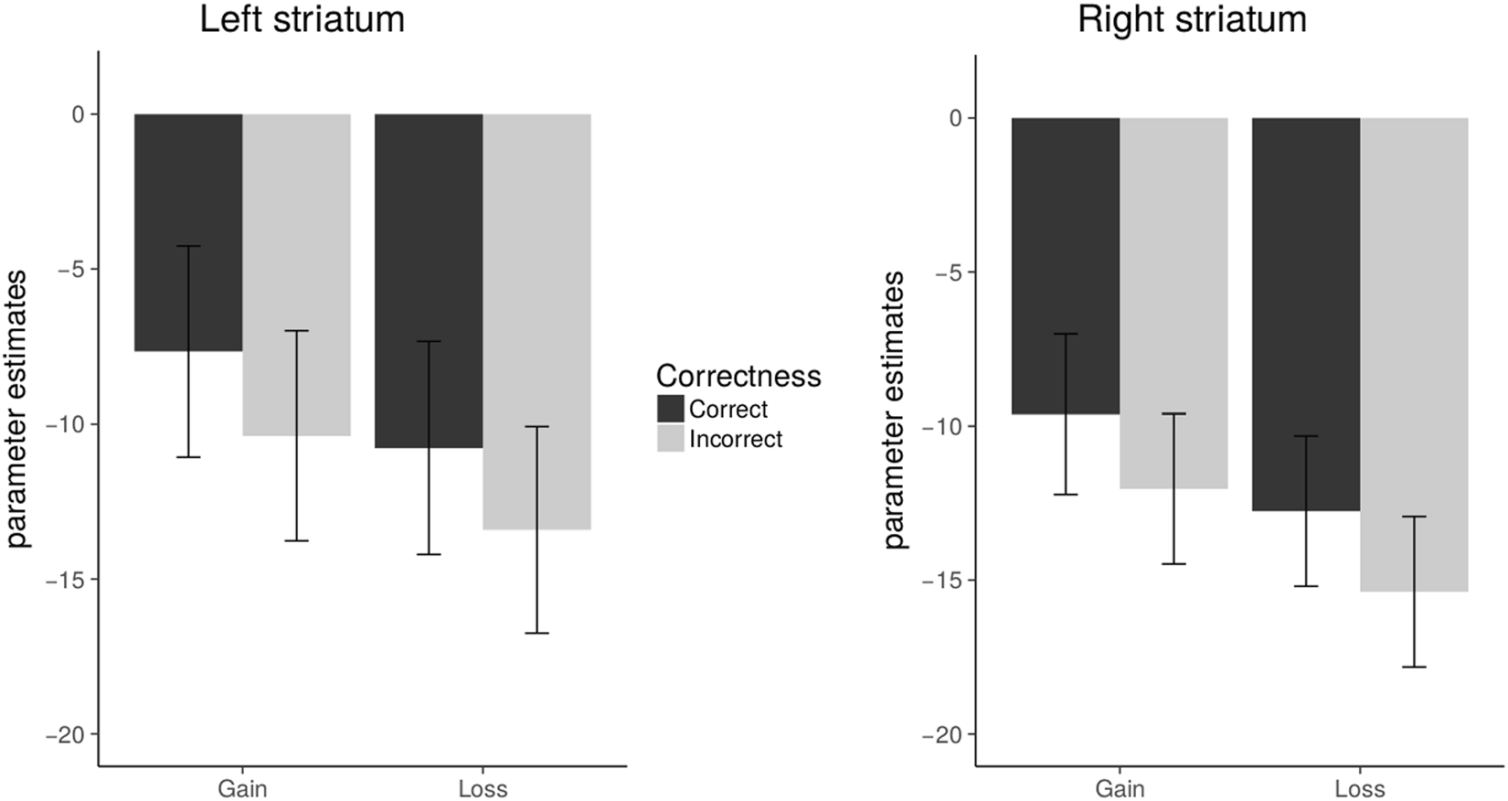
Parameter estimates of left and right anatomical striatal clusters (NAcc) for the contrasts Correct-Gain > Incorrect-Gain indicated by the label “Gain”, and Correct-Loss > Incorrect-Loss indicated by the label “Loss”.

We used parameter estimates of the left and right anatomical striatal clusters, and performed paired t-tests for Correct-Gain compared to Incorrect-Gain and Correct-Loss compared to Incorrect-Loss. Results showed significant differences for both pairs with increased striatal activation when giving the correct advice compared to incorrect advice irrespective of monetary outcome (see Fig. 4): Correct-Gain versus Incorrect-Gain (left striatum: M = 2.72, SD = 3.26, *t*(18) = 3.63, *p* = 0.002; right striatum: M = 2.42, SD = 3.08, *t*(18) = 3.42, *p* = 0.003), Correct-Loss versus Incorrect-Loss (left striatum: M = 2.64, SD = 2.17, *t*(18) = 5.30, *p* < 0.001; right striatum: M = 2.62, SD = 2.13, *t*(18) = 5.35, *p* < 0.001), and Correct-Loss versus Incorrect-Gain (left and striatum: n.s., *p*s > 0.18). The effects remained significant after Bonferroni correction.

### Exploratory analyses: the role of individual differences

#### Individual differences in confidence

To investigate whether individual differences in confidence were related to striatal activation patterns in the “I told you so” condition, we conducted correlation analyses between confidence scores (both in the Correct-Loss condition and overall scores) and the difference in striatal activation for the Correct-Loss vs. Incorrect-Loss contrast. The analyses revealed positive correlations between the striatal activations in the right hemisphere and confidence scores for both Correct-Loss (ρ = 0.654, *p* = 0.004) and overall scores (ρ = 0.630, *p* = 0.007). Left hemisphere activations did not correlate significantly with confidence scores (both *p*s > 0.25). These results suggest that individuals who were more confident in their advice (in general) showed relatively more striatal activation in response to ignored advice.

#### Individual differences in task experience

**Figure 5:**
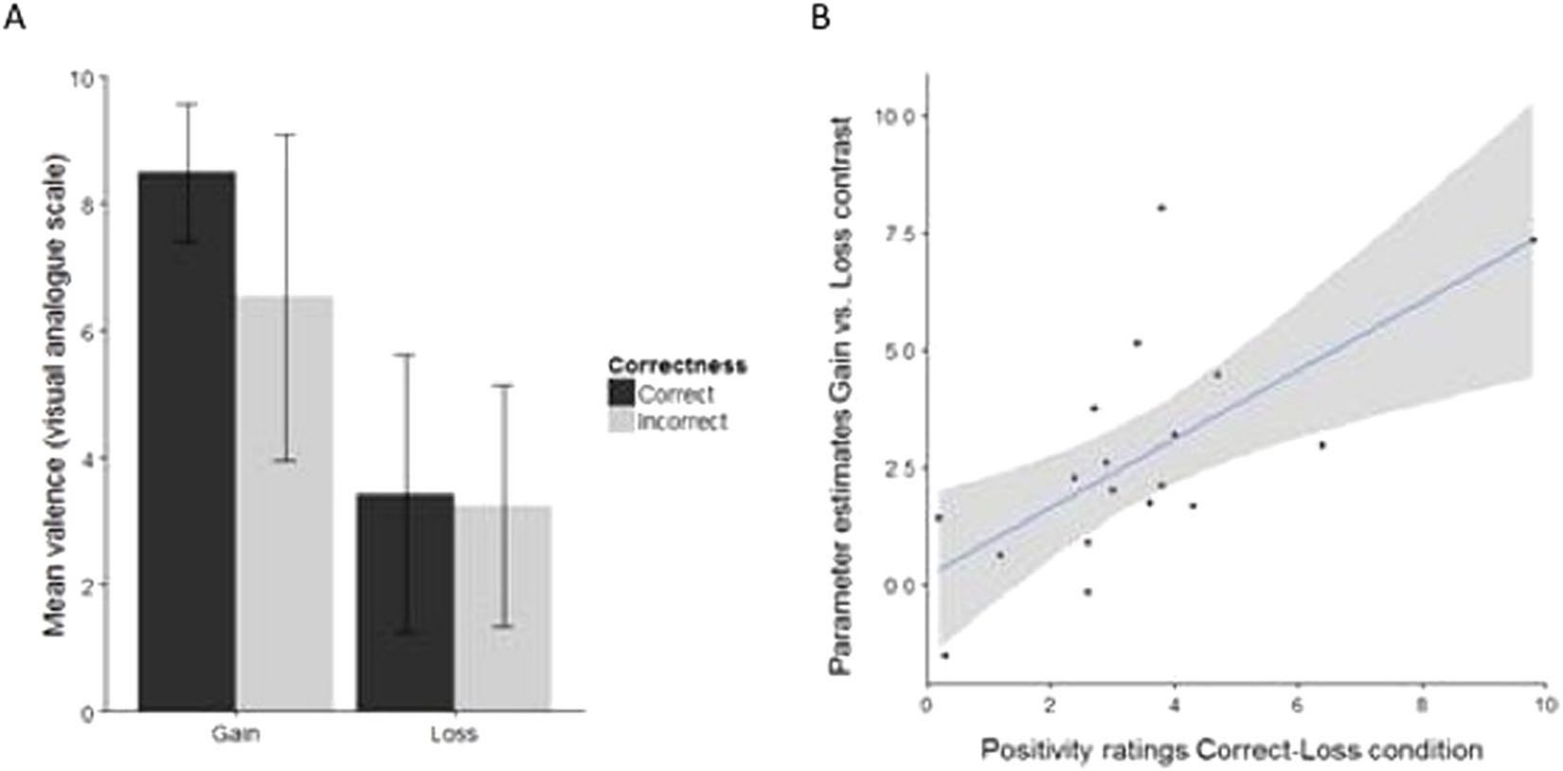
(**A**) Mean positivity ratings (ranging from 0–10) provided by the fMRI participants for the four different outcomes. Error bars depict standard deviations. (**B**) Scatterplot depicting the relationship between individual striatal activations for gain vs loss and the positivity ratings for the Correct-Loss condition (with regression line and 95% confidence band). Rating data from one participant is missing.

Additional analyses were conducted based on available exit questionnaire data collected after participants finished the fMRI study. We specifically asked participants how they experienced the four different conditions (Correct-Gain, Incorrect-Gain, Correct-Loss, and Incorrect-Loss), by having them rate the conditions on a VAS scale from very negative (0) to very positive (10). As expected, participants rated the Correct-Gain condition as most positive (see Fig. 5a) and the two conditions that resulted in a loss (Correct-Loss and Incorrect-Loss) were overall rated quite negative. The error bars in the figure represent standard deviations and it is clear that individual variance is large particularly in the conditions of Incorrect-Gain and Correct-Loss.

#### Individual differences in reward sensitivity

To investigate these individual differences further and to specifically assess whether they are related to individual differences in reward sensitivity, we conducted correlation analyses using individual parameter estimates from the structural NAcc ROI for the overall Gain vs. Loss contrast. The rationale for these analyses is that striatal activations for the overall Gain vs. Loss contrast are most likely to reflect individual reward sensitivity. The analyses revealed a strong correlation (see Fig. 5b) between the positivity ratings for Correct-Loss and the individual estimates (ρ = 0.64, *p* = 0.004 for left striatum and ρ = 0.69, *p* = 0.002 for right striatum). Please note that this correlation remains significant after removing one outlier with an almost maximal VAS score (ρ = 0.59, *p* = 0.013 for left striatum and ρ = 0.63, *p* = 0.007 for right striatum). The other three conditions did not result in significant correlations. Additionally, the self-reported feelings in the Correct-Loss condition were not significantly correlated to striatal activity in the Correct-Loss minus Incorrect-Loss contrast (both *p*s > 0.61). Importantly, this relationship shows that people who are in general more reward sensitive also experience a monetary loss as more positive when they were correct, thus suggesting that this specific condition is associated with a (subjective) feeling of reward.

#### Relationship between personality traits and neural activity

**Figure 6:**
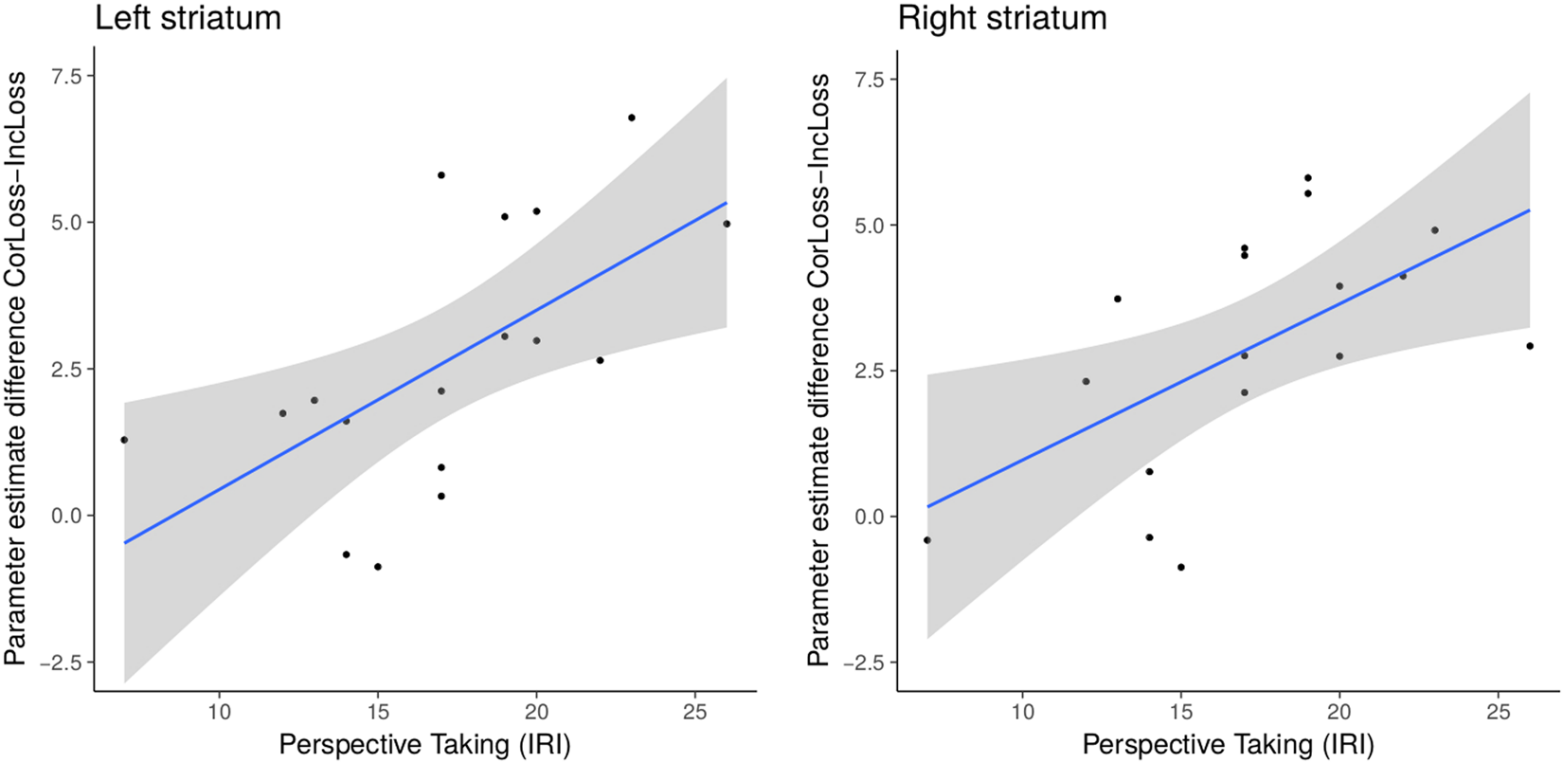
Correlation (with 95% confidence interval) between neural activation in the left and right striatum for beta-values in the contrast Correct-Loss > Incorrect-Loss and self-reported perspective taking (Interpersonal Reactivity Index). The ROI was based on the anatomical masks of the striatum (NAcc in specific) extracted from the Harvard-Oxford subcortical atlas (http://www.fmrib.ox.ac.uk/fsl/).

We also investigated whether possible individual differences in empathy (Interpersonal Reactivity Index^21^), social anxiety (Liebowitz Social Anxiety Scale*;* LSAS)^22^, psychopathic traits (Psychopathic Personality Inventory-short form; PPI-SF^23,24^), and fear of negative evaluation (FNE^25,26^) were related to striatal activation for the contrasts Correct-Gain versus Incorrect-Gain and Correct-Loss versus Incorrect-Loss (see Supplementary Materials for more details). We focused on the subscales empathic concern (measure of affective empathy; i.e. sharing feelings of others), and perspective taking of the Interpersonal Reactivity Index. Results showed a positive correlation between neural activation in the left (ρ = 0.66, *p* = 0.003) and right (ρ = 0.59, *p* = 0.012) striatum for Correct-Loss > Incorrect-Loss with perspective taking (see Fig. 6). Thus, participants who reported a higher tendency to adopt other people’s viewpoint, which is important for the understanding of other people’s emotions, showed more striatal activity when being right. None of the other correlations reached significance.

### Behavioral Control task

#### Method

For exploratory reasons, we collected additional behavioral data to gain more insight into the effects on mood/emotions for the four different conditions. For this aim, 18 participants (mean age = 23.71, 13 females) performed a modified version of the original fMRI task comprised of two blocks of 50 trials each. The task ended with an exit questionnaire about how the different outcomes were experienced: “If I was [correct/incorrect] and the other [agreed/disagreed] then I felt [satisfied/relieved/proud/disappointed/guilty]”. Participants could respond on a scale from 1 (not at all) to 7 (completely) to what extent they agreed with this statement. The questions were formulated on the basis of answers in response to an open question at the end of the fMRI experiment, where we asked participants to describe their feelings/thoughts when presented with the different outcomes (Correct-Gain, Correct-Loss, Incorrect-Gain, Incorrect-Loss). In order to test to what extent these emotions were experienced in an independent comparable sample, we decided to select the five emotions that were mentioned most frequently: satisfaction, pride, disappointment, guilt, and relief.

Participants came to the lab in pairs and practiced the task together on the same computer, taking turns in being advisor or advisee (instead of only practicing the advisor role as was the case in the fMRI study). After the practice session, they were asked to take place in separate rooms, comparable to the design used in the fMRI study. When separated, they each received an envelope informing them their role in the task (advisor or advisee). Unknown to them, both were given the role of advisor and the software simulated the role of the advisee. They then performed the task believing that they were performing it with the other participant being advisee.

#### Results

**Figure 7:**
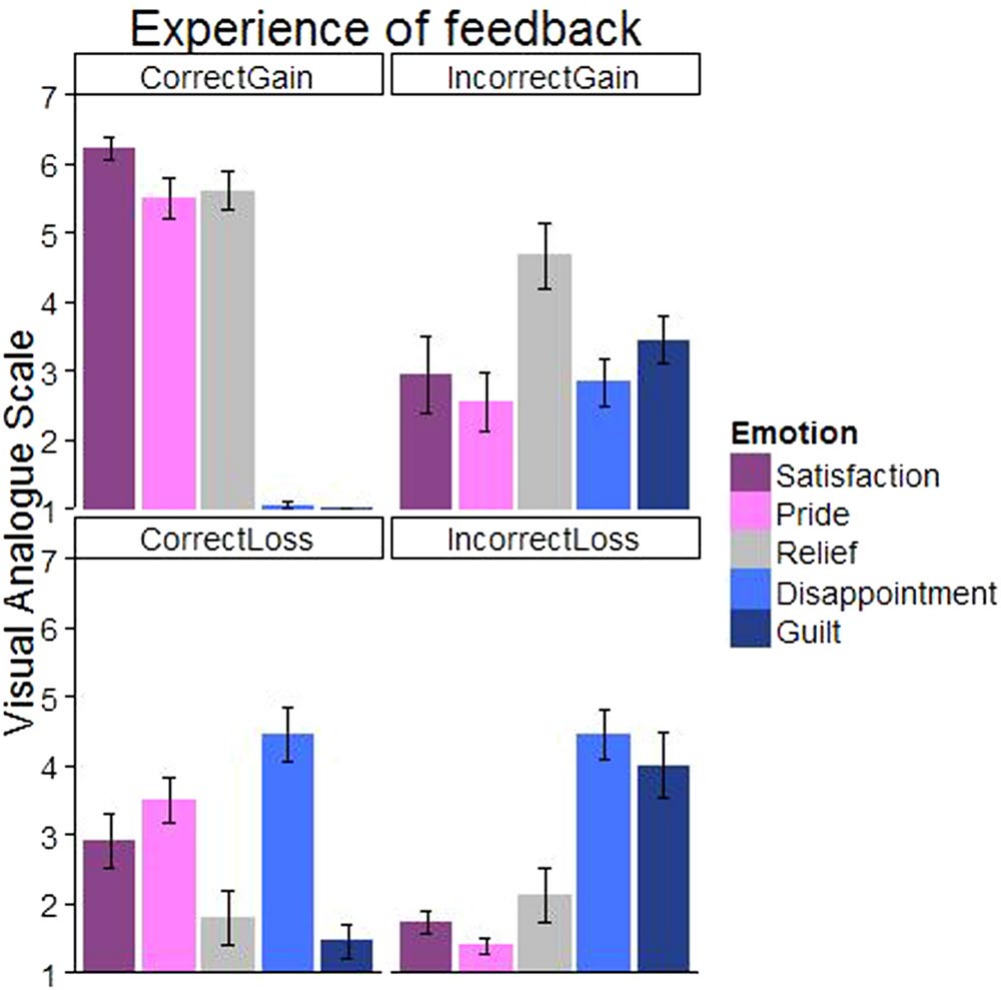
Participants reported on a visual analogue scale (1 to 7) to what extent they experienced the emotions: satisfaction, pride, relief, disappointment and guilt in the four different conditions.

Figure 7 shows the mean ratings for the five emotions and four different conditions. In order to analyze possible differences in perceived valence per condition, positive and negative valence was calculated by combining Satisfaction and Pride (positive) and Disappointment and Guilt (negative). Because relief does not have a clear association with affective valence^27^, this emotion was excluded from the following analysis.

A Valence x Correctness x Outcome ANOVA revealed a significant three-way interaction F(1,17) = 8.6, *p* = 0.009. Further exploration of this significant interaction was done using t-tests for each condition. These analyses showed main effects of Valence for Correct-Gain and Incorrect Loss. As expected, Correct-Gain is ascribed a positive valence t(34) = 24.78, *p* < 0.001, while Incorrect-Loss is associated with a negative valence, t(34) = −6.73, *p* < 0.001. Importantly, the two remaining conditions for which advices were ignored did not show any differences in perceived valence [Correct-Loss: t(34) = 0.64, *p* = 0.528, Incorrect-Gain: t(34) = −0.83, *p* = 0.411], indicating that these conditions were associated with a mix of both positive and negative feelings. It should be noted that these emotions might not give the full image as we made a selection based on subjective answers. Therefore, replication studies are necessary to confirm or expand these outcomes.

## Discussion

In the present study, we demonstrated differential striatal activations using a social visual search paradigm. We were specifically interested in an “I told you so” situation in which initial correctness of a subsequently ignored advice could be associated with an increase in reward-related brain areas even in case of a negative outcome. First, our results demonstrated enhanced striatal activation following monetary gain compared to monetary loss. Increased striatal activity was also seen for correct compared to incorrect advices. Second, a closer look at these striatal activations revealed distinctive patterns depending on the initial correctness of the advice. Direct comparisons of the situations that resulted in identical monetary outcomes showed enhanced activation in the striatum for outcomes following correct advices compared to incorrect advices. This was not only the case for situations that ended in a monetary gain, but also for those resulting in monetary loss. Finally, striatal activation patterns did not differ between the two conditions for which the participant’s advice was ignored even though one resulted in monetary gain and the other in monetary loss. The data pattern is in line with an additive-coding of gains and correct advice with largest activation (or smallest deactivation) for correct answers that resulted in a gain and largest deactivation for incorrect advice resulting in a loss. The current study therefore shows that the striatum integrates both personal performance and absolute reward outcome and may thus explain how humans can simultaneously process both positive and negative outcomes and/or emotions.

Activation in the striatum following reward outcomes is in line with previous research [see 11 for a recent review] demonstrating striatal activity in response to both social and monetary rewards^28,29^. Many prior studies showing reward-related activity included monetary incentives, predominantly because of the universality, and simplicity of this form of reward. The current study also shows that giving the correct advice is associated with increased striatal activations, which extends previous work showing that following (financial) advice activates the striatum^7,8^. Humans have a natural tendency to compare their own pay offs to those of others and studies have demonstrated a role for the striatum in a wide variety of social situations such as performing better than a co-actor (competition), winning as a team (cooperation), or observing another person getting punished because he/she violated a norm (schadenfreude)^14,30^. A reward may thus be experienced differently depending on the social context. Fliessbach and colleagues (14) compared reward-related activations for identical absolute reward outcomes with varying relative reward between the participant and a co-actor. Their results showed enhanced activation in the striatum when participants gained more money compared to their counterpart. The current work importantly extends these previous studies on social comparison by demonstrating that in the absence of clearly defined reward inequities, mere knowledge of being right modulates striatal BOLD responses to monetary losses and wins. Being right in a social context may also involve performance-based comparison processes, for example when outperforming another individual in the “I told you so” situation. Thus, the present outcomes show that giving the correct answer is not solely motivated by the desire to increase one’s merits, but also by the desire to optimize personal performance [cf. 18]. Although the underlying mechanisms are in need of further investigation, the currently found activations might reflect self-affirmation processes that are directly related to being right.

One of the limitations of the current study is that we did not include a non-social control condition in which, for example, the computer would randomly choose one of the two outcomes. The decision not to include such condition was based on the assumption that this may negatively affect the participant’s motivation to give the correct advice throughout, which is crucial for our main research aim. A consequence of not having included a non-social control condition is that we cannot rule out that effects of reward prediction errors may play a role. Incorrect responses are less frequent than correct responses – i.e. being wrong occurs in 40% of the time whereas being right around 60% –, and may therefore lead to fewer learning opportunities and differences in expectation. These frequency differences might thus serve as an alternative explanation for the overall larger striatal deactivations for the incorrect advice versus correct advice. However, we do believe that our data show some indirect support for the involvement of social reasoning in this task. Participants who score high on the perspective taking subscale of the IRI^21^ showed increased (bilateral) striatal activation when comparing losses that were preceded by correct advice to losses preceded by incorrect advice. This outcome demonstrates that participants who are more likely to take the perspective of their co-actor showed increased reward-related activations for the “I told you so” condition. We do acknowledge, however, that the lack of a good control condition limits our social interpretation of the current findings and are therefore cautious in interpreting our outcomes purely in terms of a social effect.

Another limitation is that we do not have direct evidence showing that participants really experience the “I told you so” condition as a positive, pleasant, or even rewarding event. On the contrary, valence ratings from an exit questionnaire taken immediately following the fMRI study revealed positive ratings, especially for the gain outcomes. However, large individual differences in valence descriptions were also present, except for the condition in which participants were right and gained money because the co-player followed their advice. The outcomes of the behavioral control study showed that rather than only eliciting emotions with a positive valence, the “I told you so” condition elicited a mix of both positively and negatively valenced emotions, such as “pride” and “disappointment”. This finding is in line with previous work showing that experiences of complex other-directed emotions, such as empathic care or compassion do not have a clear valence and can thus be associated with positive and negative feelings^31,32^. Additionally, one can regard the “I told you so” condition as a form of regret-related choice. Previous work has shown that activity in striatum reflects the value of an experienced outcome relative to what might have been under a different choice^33^. Interestingly, our study suggests that this counterfactual processing of a foregone reward may also hold for situations in which another person makes the final choice. However, future studies that include a social control condition are needed to confirm this.

Also, it should be noted that the striatum is not only involved in reward processing, but also plays a role in other processes such as memory^34^. To investigate if our data supports the reward-related interpretation put forward here, we focused on the individual differences in the ratings from the exit questionnaire taken immediately following the fMRI study. Analyses showed that individual positivity ratings for this condition correlated significantly with striatal activations from the overall gain versus loss contrast. The correlation between the “I told you so” ratings and striatal activity from the “I told you so” contrast was not significant, suggesting that the relation may thus not be specific to the “I told you so” condition, but rather reflects that those who report positive feelings in the “I told you so” condition have greater striatal response to gains versus losses in general. These outcomes suggest that higher levels of individual reward sensitivity (i.e., the striatal response to monetary gains versus losses) were associated with more positive experiences of the “I told you so” condition. This finding may thus support the reward-related interpretation of the currently found striatal activations, as it may reflect the subjective feeling of reward when a correct advice resulted in a loss. Moreover, although the analysis of advice confidence indicates a clear and significant correctness effect, we cannot rule out the possibility that advice confidence ratings lose value through time. Finally, our findings are based on a relatively small sample size. Therefore, future studies should aim at replicating these findings using a larger sample size including a social control condition.

To conclude, the present study highlights the strong motivational properties of being right by revealing the involvement of reward-related neural mechanisms even when the end result is a monetary loss. Additionally, the current study suggests a role for individual differences in emotional valence specifically when processes tend to be more complicated because of the mix of personal performance and monetary outcome. In future studies, it will be important to further explore these social reward processes ideally in larger sample sizes, including the role of emotional valence, individual differences, and in comparison with a non-social control condition.

## Electronic supplementary material

Supplementary information
