# Supplementary material for: Being right, but losing money: the role of striatum in joint decision making
Source: Sci Rep. 2018 Apr 30;8:6711. doi: 10.1038/s41598-018-24617-3 (PMC5928107; doi:10.1038/s41598-018-24617-3)
Supplement: Supplementary file 1 — Supplementary information [file 41598_2018_24617_MOESM1_ESM.doc]

RUNNING HEAD: BEING RIGHT IS REWARDING

Being right, but losing money: the role of striatum in joint decision making

Ruissen, M. I.^a,b,1^, Overgaauw, S.^a,b,*,1^, & de Bruijn, E. R. A.^a,b^

^a^Institute of Psychology, Leiden University, The Netherlands

^b^Leiden Institute for Brain and Cognition (LIBC), Leiden, The Netherlands

*Corresponding author. Address: Faculty of Social Sciences, Leiden University,

Wassenaarseweg 52, 2333AK Leiden, The Netherlands.

E-mail address: s.overgaauw.2@fsw.leidenuniv.nl (S. Overgaauw).

^1^ Both authors contributed equally.

**Supplementary materials**

*S1. Trait questionnaires used for exploratory analyses*

*Interpersonal Reactivity Index (IRI; [19]).* Two scales of the IRI were obtained: empathic concern (e.g., ‘I am often quite touched by things that I see happen’; 7 items), and perspective taking (e.g., ‘I try to look at everybody's side of a disagreement before I make a decision’; 7 items). Each item was rated on a 5-point Likert scale with 0) completely untrue, 1) not quite true, 2) in between, 3) quite true and 4) completely true. All questions were (re) scored such that higher scores reflected higher empathy. Sum scores were calculated per scale.

*Liebowitz Social Anxiety Scale (LSAS; [20]).* Participants completed the 24-item LSAS consisting of the subscales fear (12 items, rated on a four-point scale with 0 = none, 1 = mid, 2 = moderate, 4 = severe) and avoidance (12 items, rated on a four-point scale with 0 = never, 1 = occasionally, 2 = often, 4 = usually) of social situations (e.g. Eating in public places). Sum scores were calculated with higher scores indicating higher social anxiety.

*Psychopathic Personality Inventory-short form (PPI-SF; [21,22]).* To assess psychopathic traits, participants were asked to complete the 100-item PPI-SF. The PPI-SF contains 8 subscales and had to be answered on a 4-point Likert scale (1 = false and 4 = true). Sum scores were calculated with higher scores indicating higher psychopathic traits.

*Fear of negative social evaluation (FNSE; [23,24].* Self-reported fear of negative evaluation was assessed using 4-items of the Interpersonal Sensitivity Measure [23]. Participants were asked to indicate to what extent the statements applied to them on a 4-point scale with answers varying from 1) not quite true to 4) completely true. Sum scores were calculated; higher scores indicated higher fear of negative evaluations.

Please note that questionnaire data was not available for two participants and one additional participant did not complete the PPI-SF.

*S2. Individual parameter estimates*


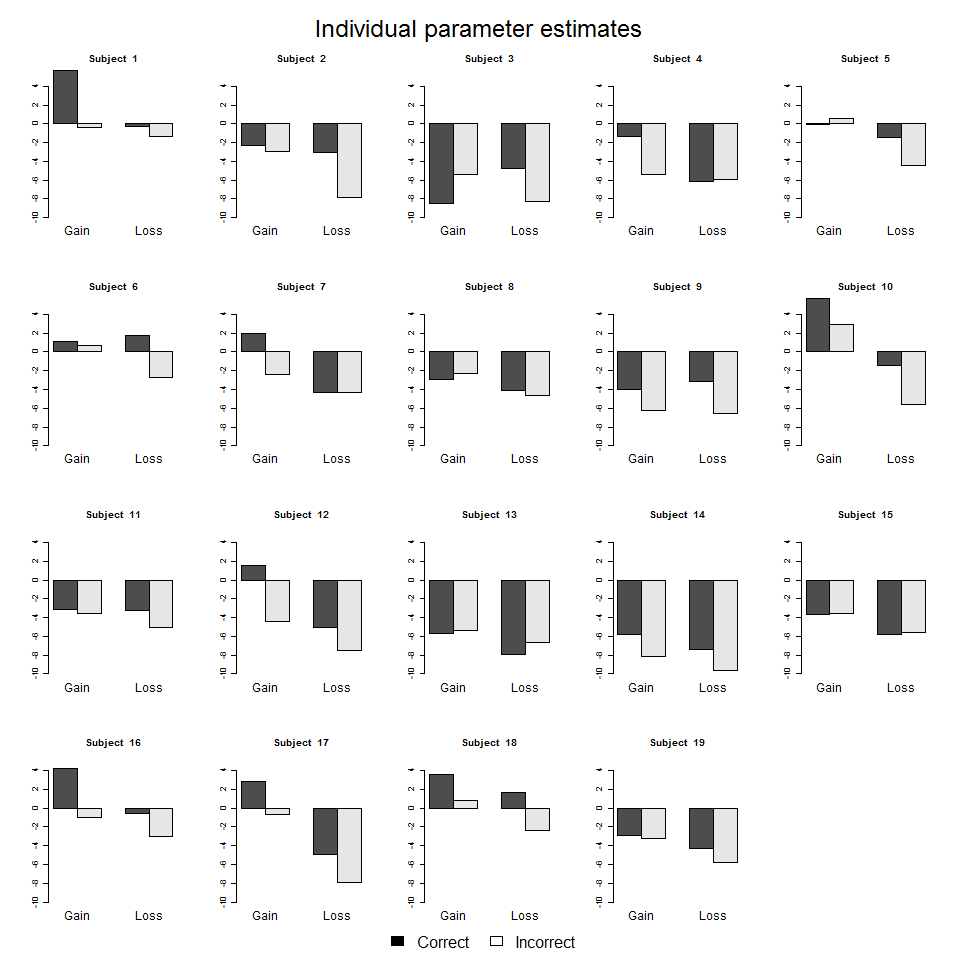


Figure S1. Mean parameter estimates for left and right striatum for each individual subject as function of Correctness and Outcome.

S3. *parametric analyses with confidence level as a trial-by-trial regressor*

We ran first level parametric analyses with Confidence level, Correctness, and Outcome (all modulators were demeaned on a subject level) as trial-by-trial regressors without preselected conditions using Outcome onset. We found bilateral striatal activity and medial prefrontal cortex activity for Correctness (FWE-corrected, *p* < .05; see Figure S2). For Outcome onset, we found activation in the right and left caudate and in the cuneus (FWE-corrected, *p* < .05; see Figure S3).


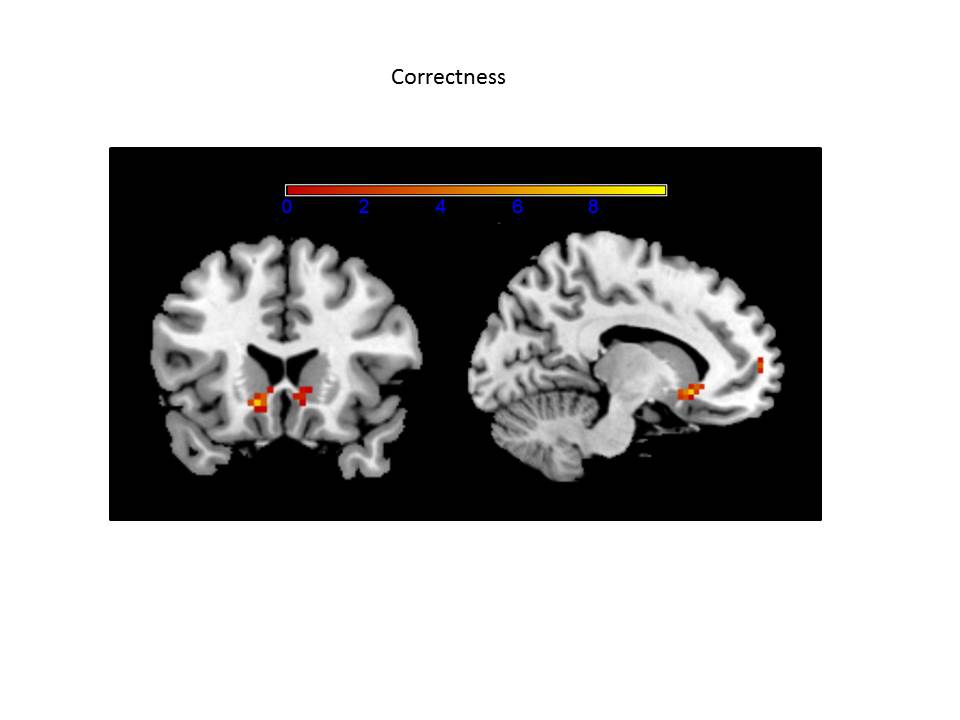


Figure S2. First level parametric analysis with Correctness (demeaned on a subject level) as a trial-by-trial regressor without preselected conditions using Outcome onset resulted in bilateral striatal activity and medial prefrontal cortex activity (FWE-correction; *p* < .05).


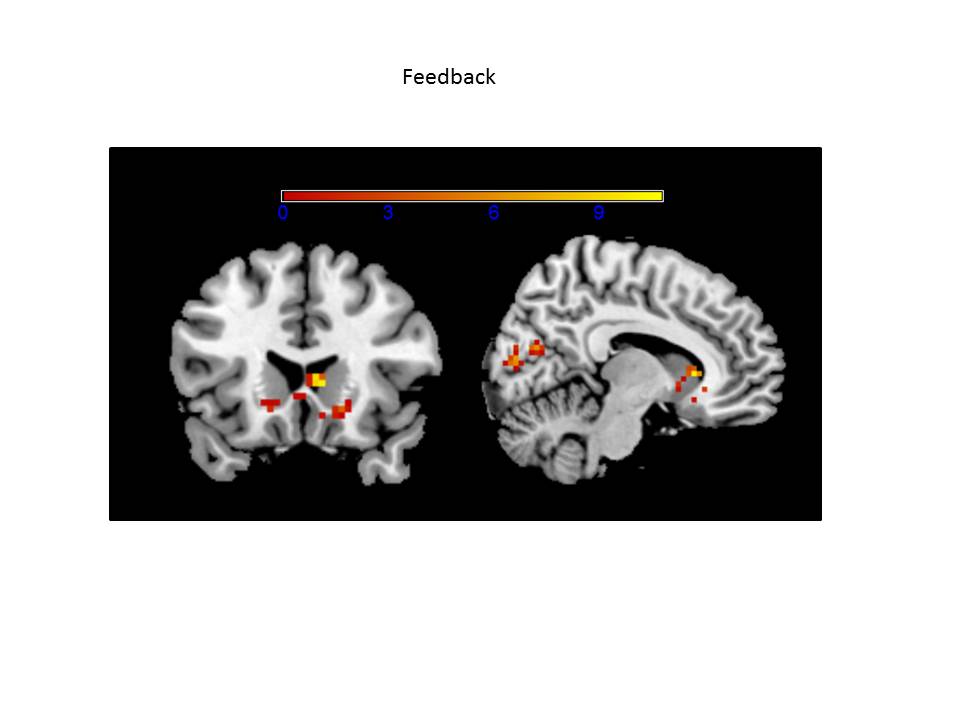


Figure S3. First level parametric analysis with Outcome (demeaned on a subject level) as a trial-by-trial regressor without preselected conditions using Outcome onset resulted in bilateral caudate and cuneus activity (FWE-correction; *p* < .05).

S4. *Individual differences in reward sensitivity*


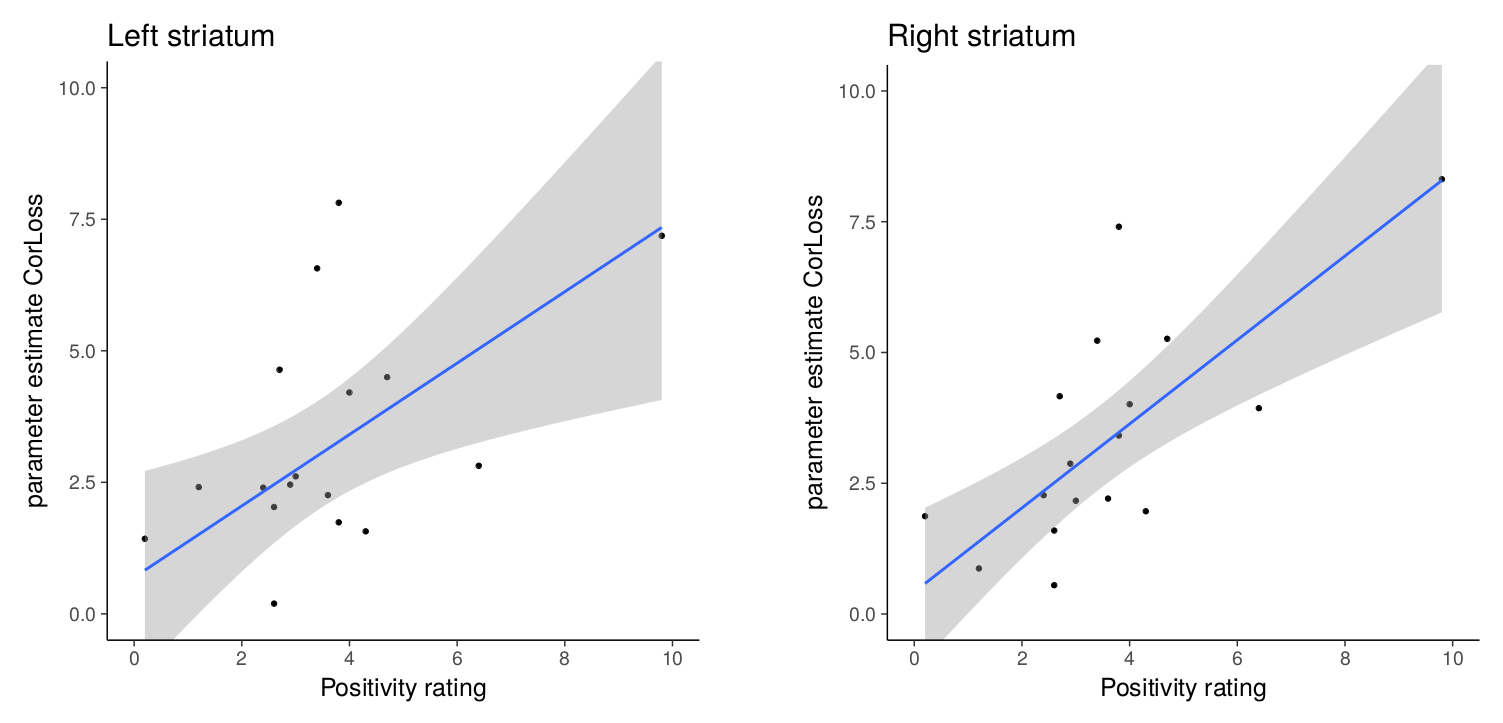


Figure S4. Correlation (with 95% confidence interval) between neural activation in the left and right striatum for beta-values in the contrast Correct-Loss > Incorrect-Loss and self-reported positivity ratings for the Correct-Loss condition.
